# Chlorination Disinfection By-Products in Drinking Water and Congenital Anomalies: Review and Meta-Analyses

**DOI:** 10.1289/ehp.0900677

**Published:** 2009-06-15

**Authors:** Mark J. Nieuwenhuijsen, David Martinez, James Grellier, James Bennett, Nicky Best, Nina Iszatt, Martine Vrijheid, Mireille B. Toledano

**Affiliations:** 1 Centre for Research in Environmental Epidemiology, Barcelona, Spain; 2 Municipal Institute of Medical Research (IMIM-Hospital del Mar), Barcelona, Spain; 3 CIBER Epidemiologia y Salud Pública, Barcelona, Spain; 4 Imperial College London, London, United Kingdom

**Keywords:** birth defects, congenital anomalies, disinfection by-products, fetal development, reproductive health, trihalomethanes

## Abstract

**Objectives:**

The aim of this study was to review epidemiologic evidence, provide summary risk estimates of the association between exposure to chlorination disinfection by-products (DBPs) and congenital anomalies, and provide recommendations for future studies.

**Data sources and extraction:**

We included all published epidemiologic studies that evaluated a relationship between an index of DBP exposure (treatment, water source, DBP measurements, and both DBP measurements and personal characteristics) and risk of congenital anomalies. When three or more studies examined the same exposure index and congenital anomaly, we conducted a meta-analysis to obtain a summary risk estimate comparing the highest exposure group with the lowest exposure group. When five or more studies examined total trihalomethane (TTHM) exposure and a specific congenital anomaly, we conducted a meta-analysis to obtain exposure–response risk estimates per 10 μg/L TTHM.

**Data synthesis:**

For all congenital anomalies combined, the meta-analysis gave a statistically significant excess risk for high versus low exposure to water chlorination or TTHM [17%; 95% confidence interval (CI), 3–34] based on a small number of studies. The meta-analysis also suggested a statistically significant excess risk for ventricular septal defects (58%; 95% CI, 21–107), but this was based on only three studies, and there was little evidence of an exposure–response relationship. We observed no statistically significant relationships in the other meta-analyses. We found little evidence for publication bias, except for urinary tract defects and cleft lip and palate.

**Conclusion:**

Although some individual studies have suggested an association between chlorination disinfection by-products and congenital anomalies, meta-analyses of all currently available studies demonstrate little evidence of such an association.

Disinfection of drinking water has led to a major improvement in public health since it was first applied in the 20th century. It has now been > 30 years since it was discovered that by-products can be formed in small quantities as part of the chlorination process (Rook 1974). Chlorination disinfection byproducts (DBPs) are formed when water is chlorinated and organic matter in the water reacts with chlorine. Their formation and occurrence depends on many factors, including the chlorine dose, type of treatment, pH, temperature, residence time, and bromine levels [International Programme on Chemical Safety (IPCS) 2000; Nieuwenhuijsen et al. 2000a]; up to 600 DBPs have been identified (Richardson 1998; Richardson et al. 2008). Different mixtures of by-products may exist in different locations, depending on the various factors mentioned above, making it more difficult to assess any health effects of DBPs, particularly in epidemiologic studies.

Ingestion of water is thought to be the main route of exposure for nonvolatile DBPs such as haloacetic acids (HAAs). Exposure to volatile DBPs such as trihalomethanes (THMs) may occur through inhalation and absorption during activities such as showering, bathing, and swimming (Nieuwenhuijsen et al. 2000a). Modeling of THM uptake has suggested that swimming may lead to the highest levels of THMs in the blood (Whitaker et al. 2003).

Various reviews have been conducted on the epidemiology of chlorination by-products and reproductive outcomes. These have concluded that there are still many problems to overcome and that the results are inconsistent and inconclusive, except perhaps for small-for-gestational-age/intrauterine growth retardation (Bove et al. 2002; Gevecker Graves et al. 2001; IPCS 2000; Nieuwenhuijsen et al. 2000b; Reif et al. 1996; Tardiff et al. 2006). In addition, two meta-analyses by Hwang and Jaakkola (2003) and Hwang et al. (2008) reported evidence for an effect of exposure to chlorination by-products on the risk of neural tube defects, urinary system defects, and ventricular septal defects, but risks for other anomalies were considered heterogeneous and inconclusive. However, the meta-analyses by Hwang and Jaakkola (2003) included only five studies and did not include more recent studies, and the study by Hwang et al. (2008) generally focused on a few subcategories of anomalies and also did not include some recent studies. In this article we review the epidemiologic evidence and provide summary risk estimates of the association between chlorination disinfection by-products and congenital anomalies; we also provide recommendations for future studies.

## Methods

We searched PubMed (National Center for Biotechnology Information 2009) using the following key words: “chlorination,” “chlorination by-products,” “chlorination byproducts,” “trihalomethanes,” and “haloacetic acids.” We searched for these key words alone and in combination with the following key words for congenital anomalies: “birth defects,” “congenital anomalies,” “congenital malformations,” “urinary,” “respiratory,” “cardiovascular,” “neural tube defects” (NTDs), and “cleft lip/palate.” Furthermore, we searched in review and meta-analysis articles on the topic (Bove et al. 2002; Gevecker Graves et al. 2001; Hwang and Jaakkola 2003; Hwang et al. 2008; IPCS 2000; Nieuwenhuijsen et al. 2000b; Reif et al. 1996; Tardiff et al. 2006). We retrieved all epidemiologic studies examining the relation between chlorination byproducts and congenital anomalies.

We included all epidemiologic studies that evaluated the relationship between some index of chlorination by-products (treatment, water source, DBP measurements, and both DBP measurements and personal characteristics) and any congenital anomalies. We did not score the studies for quality, given the relatively small number of studies involved.

We obtained summary risk estimates for general water chlorination by conducting meta-analyses when three or more studies featured the same congenital anomalies. In these analyses we calculated risk estimates for high versus low exposure, using the exposure groups as defined in the original studies [e.g., high vs. low total trihalomethane (TTHM)] concentration, chlorination vs. nonchlorination, or chlorination vs. chloramination]. When three or more studies had measurements of individual THMs or brominated fractions, we conducted meta-analyses, again comparing the highest versus the lowest exposure groups of the original studies. We tested for heterogeneity in risk estimates using the *Q* test (Cochran 1954). When the result of the *Q* test was statistically significant (*p* < 0.05), implying heterogeneity between the studies, we used random effects analyses, using the method of DerSimonian and Laird (1986). When the *Q* test was not statistically significant, we used fixed effects analyses, using the Mantel-Haenszel method (Mantel and Haenszel 1959). The summary estimates were weighted by the inverse variance of each study, taking into account whether a fixed or random effects model was used. We used STATA statistical software (StataCorp, College Station, TX, USA).

Where five or more studies, each with the same outcome and exposure classification (applicable only to TTHM) were split into three or more exposure categories, we conducted a meta-analysis to obtain exposure–response risk estimates per 10 mg/L TTHM. All the included studies provided data on exposure in a categorical way. The midpoint of each given exposure category was used in the estimation of exposure–response slopes for each of the studies. If the highest exposure category was open-ended (i.e., greater than some concentration), a value of half the width of the previous exposure category was added to the cut point of highest exposure category, using the method described by Key et al. (2006). In the absence of data to the contrary, we assumed that the natural logarithm of the measure of effect varied linearly with concentration of TTHM in drinking water. Exposure–response slopes for those exposed to TTHM in drinking water were calculated using linear regression of log-transformed risk estimates. We performed the meta-analyses using R software (R Project for Statistical Computing 2009) and scripts adapted from those of Key et al. (2006). A random effects model was used for the meta-analysis of exposure–response slopes of individual studies. Regression slopes of exposure–response slopes derived from individual studies were plotted together with the summary slope produced from their meta-analysis.

We also produced forest and funnel plots (Light and Pillemer 1984). Forest plots show the odds ratio (OR) with its confidence interval (CI) for each study included in the meta-analyses and the estimation of the summary OR. The sizes of the square markers of OR in the plot represent the relative weight each study contributed to the regression.

Funnel plots show, for every study, the effect estimate (OR and relative risk) of the study against some precision measure (SE in this case) to examine possible bias due to publication. We also conducted a weighted Egger test, a linear regression in which the response is the estimated effect and the explanatory variable is a precision term (1/SE). A large deviation from zero of the slope term suggests publication bias (Egger et al. 1997).

## Results

The extracted and evaluated studies are shown in Table 1. A third of these studies were conducted in the United States (Aschengrau et al. 1993; Bove et al. 1995; Klotz and Pyrch 1999; Luben et al. 2008; Shaw et al. 2003), followed by Sweden (Cedergren et al. 2002; Källen and Robert 2000), England and Wales (Iszatt N, Nieuwenhuijsen MJ, Toledano MB, Nelson P, Elliott P, unpublished data; Nieuwenhuijsen et al. 2008), Canada (Dodds et al. 1999; Dodds and King 2001), Norway (Hwang et al. 2002; Magnus et al. 1999), Australia (Chisholm et al. 2008), and Taiwan (Hwang et al. 2008). Five were case–control studies [Aschengrau et al. 1993; Iszatt N, Nieuwenhuijsen MJ, Toledano MB, Nelson P, Elliott P, unpublished data; Klotz and Pyrch 1999; Luben et al. 2008; Shaw et al. 2003 (includes two studies)], whereas the rest were cross-sectional, population-based studies, generally using registry data, which limited the opportunity to adjust for potential confounders. Most included live births, fetal deaths, and terminations. A number of the studies used only treatment method (chlorination vs. non-chlorination, chlorination vs. chloramination) as the index of exposure (Aschengrau et al. 1993; Hwang et al. 2002; Källen and Robert 2000; Magnus et al. 1999), whereas the other studies used a measure of DBP exposure level, mostly THMs (Bove et al. 1995; Cedergren et al. 2002; Chisholm et al. 2008; Dodds et al. 1999; Dodds and King 2001; Iszatt N, Nieuwenhuijsen MJ, Toledano MB, Nelson P, Elliott P, unpublished data; Klotz and Pyrch 1999; Luben et al. 2008; Nieuwenhuijsen et al. 2008; Shaw et al. 2003). Generally, studies used TTHM levels, but some studies also evaluated individual THMs or the brominated fraction (Dodds and King 2001; Iszatt N, Nieuwenhuijsen MJ, Toledano MB, Nelson P, Elliott P, unpublished data; Nieuwenhuijsen et al. 2008; Shaw et al. 2003). The levels of brominated THM were high in Australia and made up most of the TTHM there (Chisholm et al. 2008). Klotz and Pyrch (1999) also had a measure of haloacetonitrile (HAN) and HAA exposure, and Luben et al. (2008) a measure of HAA exposure. Levels of DBPs in Scandinavia (Cedergren et al. 2002; Hwang et al. 2002; Källen and Robert 2000; Kuivinen and Johnsson 1999; Magnus et al. 1999) and Taiwan (Hwang et al. 2008) tended to be lower than in the other countries, with median levels around 10 μg/L TTHM, with approximately 20% > 20 μg/L. The Canadian (Dodds et al. 1999; Dodds and King 2001) and Australian (Chisholm et al. 2008) studies examined the highest TTHM levels (> 100 μg/L). Three studies examined personal behavioral activities such as ingestion, showering, and bathing (Iszatt N, Nieuwenhuijsen MJ, Toledano MB, Nelson P, Elliott P, unpublished data; Klotz and Pyrch 1999; Luben et al. 2008). The largest study was conducted in England and Wales and included > 2.5 million births and > 20,000 cases of congenital anomalies (Nieuwenhuijsen et al. 2008). Dodds et al. (1999) and Dodds and King (2001) used the same subjects but examined the effect of TTHM and individual THMs, respectively. The study by Hwang et al. (2002) included 3 years of subjects from Magnus at al. (1999), and therefore only the former was included in any of the meta-analyses. The report by Shaw et al. (2003) contained two case–control studies (study 1 and study 2), and they have been treated as separate studies.

**Figure 1:**
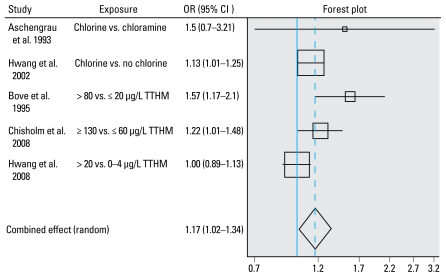
Study and summary risk estimates for any congenital anomalies and chlorination by-products. Test for heterogeneity: *Q* = 9.944 on four degrees of freedom (*p*= 0.041). Egger test: weighted *p*-value for intercept 0.16.

A number of studies found statistically significant positive associations between water chlorination (THM levels or chlorinated water) and any congenital anomaly (Bove et al. 1995; Chisholm et al. 2008; Hwang et al. 2002), whereas others did not (Aschengrau et al. 1993; Hwang et al. 2008; Magnus et al. 1999). There was evidence of heterogeneity between studies (*p* = 0.041). The meta-analysis, using a random effects model, produced a statistically significant summary effect measure of 17% excess risk (95% CI, 2–34) [(Table 2, Figure 1; see also Supplemental Material 1, Table 1, available online (doi:10.1289/ehp. 0900677.S1 via http://dx.doi.org/)].

Neural tube defects were one of the most commonly studied groups of congenital anomalies, and three studies found statistically significant positive associations between THM levels or chlorinated water and neural tube defects (Bove et al. 1995; Dodds and King 2001; Klotz and Pyrch 1999), whereas others did not (Chisholm et al. 2008; Dodds et al. 1999; Hwang et al. 2002; Källen and Robert 2000; Magnus et al. 1999; Nieuwenhuijsen et al. 2008; Shaw et al. 2003) [Table 2; see also Supplemental Material 1, Tables 2a–2d (doi:10.1289/ehp.0900677.S1)]. There appeared to be little difference between the findings of using TTHM levels or brominated species as the exposure index. The meta-analyses for high versus low exposure for water chlorination (THM levels or chlorinated water) and bromodichloromethane (BDCM) showed some excess of risk, but it was not statistically significant. We found no evidence for an exposure–response relationship with TTHM levels (OR = 1.00; 95% CI, 0.94–1.07 per 10 μg/L TTHM) [see Supplemental Material 3, Figures 1–3 (doi:10.1289/ehp.0900677.S1)]. Klotz and Pyrch (1999) found a statistically significant association between TTHM levels in the water and neural tube defects, but not with levels of haloacetonitriles and haloacetates. Inclusion of personal behavioral activities made little difference in the results. In addition, the effects were most pronounced in offspring from women who did not take supplementary vitamins, but these findings were not confirmed by Shaw et al. (2003). Findings of the subcategories of neural tube defects including anencephalus, spina bifida, and hydrocephalus were also mixed, and none of the studies found a statistically significant association except for Shaw et al. (2003) who found a statistically significant reduced risk. The meta-analyses showed some excess of risk for anencephalus and spina bifida, but it was not statistically significant.

**Figure 2:**
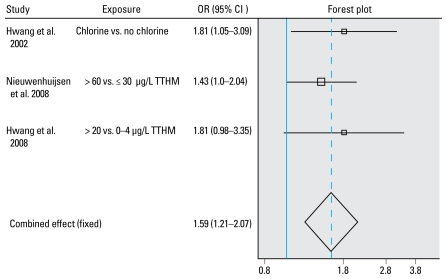
Study and summary risk estimates for ventricular septal defects and chlorination by-products. Test for heterogeneity: *Q* = 0.732 on two degrees of freedom (*p*= 0.69). Egger test: weighted *p*-value for intercept 0.13.

Cedergren et al. (2002), Chisholm et al. (2008), and Hwang et al. (2002) found statistically significant positive associations between chlorinated water with a higher color content, levels of TTHM > 10 μg/L, and high levels of DBPs (≥ 130 μg/L), respectively, and cardiovascular congenital anomalies, but other studies did not observe such an association (Bove et al. 1995; Dodds et al. 1999; Dodds and King 2001; Källen and Robert 2000; Magnus et al. 1999; Nieuwenhuijsen et al. 2008; Shaw et al. 2003) [Table 2; see Supplemental Material 1, Tables 3a–3c (doi:10.1289/ehp.0900677.S1)]. There was some evidence for heterogeneity in study results (*p* = 0.017). The random effects estimate of the meta-analysis for all major cardiac defects combined showed a nonstatistically significant 16% excess risk. There was no evidence of an exposure–response relationship (OR = 1.01; 95% CI, 0.95–1.08 per 10 μg/L TTHM) [see Supplemental Material 3, Figures 1–3 (doi:10.1289/ehp.0900677.S1)]. Furthermore, the brominated species did not show statistically significant associations. However, in a very large study, Nieuwenhuijsen et al. (2008) found a statistically significant association between bromoform levels and a subset of isolated major cardiovascular defects [2 to < 4 vs. < 2 μg/L bromoform, OR = 1.13 (95% CI, 0.99–1.29); ≥ 4 vs. < 2 μg/L bromoform, OR = 1.18 (95% CI, 1.00–1.39)]. Furthermore, ventricular septal defects showed an increased risk for high versus low exposure in all three studies examining these defects (Hwang et al. 2002, 2008; Nieuwenhuijsen et al. 2008), and a statistically significant excess risk was observed in the meta-analysis (OR = 1.59; 95% CI, 1.21–2.07) (Figure 2). However, only Hwang et al. (2002) showed some indication of an exposure–response relationship. Ventricular septal defects showed little association with brominated THM levels in the study by Nieuwenhuijsen et al. (2008). No statistically significant associations were observed with atrial septal defects in the two studies by Hwang et al. (2002, 2008).

Two studies found a statistically significant positive association between chlorinated water and congenital anomalies of the respiratory system (Aschengrau et al. 1993; Hwang et al. 2002), but two other studies using TTHM levels did not (Chisholm et al. 2008; Nieuwenhuijsen et al. 2008) [Table 2; see also Supplemental Material 1, Table 4 (doi:10.1289/ehp.0900677.S1)]. Results of the two Swedish studies were inconsistent (Hwang et al. 2002; Magnus et al. 1999). The meta-analysis showed some excess of risk, but it was not statistically significant.

Studies on oral cleft or/and cleft palate have generally shown no statistically significant associations with chlorinated water, TTHM, and brominated THMs, except for the study by Bove et al. (1995) [Table 2; see also Supplemental Material 1, Tables 5a,5b (doi:10.1289/ehp.0900677.S1)]. The meta-analyses showed no evidence for an association. In addition, the exposure–response relationship was not statistically significant (OR = 1.02; 95% CI, 0.97–1.06 per 10 μg/L TTHM) [see Supplemental Material 3, Figures 1–3 (doi:10.1289/ehp.0900677.S1)].

Two studies reported statistically significant associations between chlorinated water or TTHM levels and urinary system defects (Aschengrau et al. 1993; Magnus et al. 1999), whereas one did not (Nieuwenhuijsen et al. 2008) [Table 2; see also Supplemental Material 1, Tables 6a,6b (doi:10.1289/ehp.0900677.S1)]. Chisholm et al. (2008) reported almost statistically significant effects (OR = 1.40; 95% CI, 0.98–1.99), as did Hwang et al. 2002 (OR = 1.46; 95% CI, 1.00–2.13), and there was evidence for heterogeneity (*p* = 0.012) in study results. Nieuwenhuijsen et al. (2008) did not show an association with brominated THM species and found no statistically significant associations for obstructive urinary defects, although Hwang et al. (2002, 2008) did find excess risk. The meta-analyses showed some excess of risk, but it was not statistically significant.

Two studies examined the association between abdominal wall defects and chlorinated water or THM levels and did not find an association (Källen and Robert 2000; Nieuwenhuijsen et al. 2008) [see Supplemental Material 1, Table 7 (doi:10.1289/ehp. 0900677.S1)]. However, Nieuwenhuijsen et al. (2008) found a statistically significant association between isolated gastroschisis and bromoform levels [2–4 vs. < 2 μg/L bromoform, OR = 1.11 (95% CI, 0.85–1.45); ≥ 4 vs. < 2 μg/L bromoform, OR = 1.38 (95% CI, 1.00–1.92)].

The studies on hypospadias generally showed no statistically significant associations between chlorinated water or TTHM levels, and neither did the meta-analysis [Table 2; see also Supplemental Material 1, Table 8 (doi:10.1289/ehp.0900677.S1)]. However, Iszatt et al. (Iszatt N, Nieuwenhuijsen MJ, Toledano MB, Nelson P, Elliott P, unpublished data) reported a statistically significant excess risk between BDCM ingestion estimates and hypospadias (1st vs. 4th quartile BDCM, OR = 1.69; 95% CI, 1.04–2.73). Similarly, Luben et al. (2008) reported that risk of hypospadias was associated with moderate ingestion of > 0–32.5 μg/day TTHM. Luben et al. (2008) did not show a statistically significant association between HAA exposure levels and hypospadias but showed excess risk for TTHM uptake, which takes into account various personal behaviors and exposure routes, although this was not statistically significant. Iszatt et al. (Iszatt N, Nieuwenhuijsen MJ, Toledano MB, Nelson P, Elliott P, unpublished data) did not find such an association in a much larger study.

Generally there was little evidence for publication bias, except for cleft lip and palate, specifically when comparing the exposure–response relationships (*p* = 0.04), and urinary tract defect (*p* = 0.002) [Table 2; see also Supplemental Material 2, Figures 1–15 (doi:10.1289/ehp.0900677.S1)]. However, it should be noted that publication bias tests and funnel plots are not considered rigorous tests when the number of studies is small.

## Discussion

The epidemiologic studies we reviewed showed inconsistent results for an association between drinking water chlorination by-products and risk of all congenital anomalies combined and of specific groups of anomalies. For all congenital anomalies combined, the meta-analysis gave a statistically significant excess risk for high versus low exposure to water chlorination or TTHM (17%; 95% CI, 3–34), based on a small number of studies. The meta-analysis also suggested a statistically significant excess risk for ventricular septal defects (58%; 95% CI, 21–107), but this was based on only three studies, and there was little evidence of an exposure–response relationship. We observed no statistically significant relationships in the other meta-analyses. We found little evidence for publication bias except for urinary tract defects and cleft lip and palate.

Major limiting factors in studies on chlorination by-products and congenital anomalies are crude exposure assessment (with the exception of more recent studies), small samples sizes, heterogeneity in outcomes, and, to a lesser extent, potential for bias and confounding and ability to detect susceptible groups. These factors together may explain some of the mixed results and possibly the lack of associations. Furthermore, combined with the small number of studies included in the meta-analyses, these factors also reduce the strength of any conclusions that can be drawn from meta-analyses. These analyses are therefore not meant to provide final conclusions on the subject, but instead evaluate the current status of this growing body of research and offer guidance for the way forward.

Use of ecologic water supply zone estimates as an exposure index may result in exposure misclassification (Whitaker et al. 2003). Furthermore, ingestion has generally served as the primary exposure route of interest, in spite of the fact that uptake through showering, bathing, and swimming could be considerable, specifically for THMs; such exposure has been considered in only a few studies (e.g., Iszatt N, Nieuwenhuijsen MJ, Toledano MB, Nelson P, Elliott P, unpublished data; Klotz and Pyrch 1999; Luben et al. 2008). Combining information on individual water use with water zone estimates could provide better exposure estimates, but the individual information should be evaluated for measurement error, because within-subject variability in questionnaire data may be substantial (Forssén et al. 2008) and attenuate risk estimates. Furthermore, exposure estimates have been based primarily on maternal residence at birth. This ignores any exposure that occurs outside the home (e.g., in the workplace) and also ignores the possibility that a mother has moved to another residence during her pregnancy. Therefore, exposure assessment based on maternal residence at birth may result in exposure misclassification. In addition, studies from Scandinavia (Cedergren et al. 2002; Hwang et al. 2002; Källen and Robert 2000; Magnus et al. 1999) and Taiwan (Hwang et al. 2008), for example, have generally shown low levels of DBPs with a small range, making risk estimation more difficult because of a higher probability of exposure misclassification, particularly where seasonal variability has not been taken into account. On the whole, epidemiologic studies have used THMs as a proxy for total DBP load, but THMs are not necessarily a good proxy measure. Only two studies have investigated other DBPs such as HAAs (Klotz and Pyrch 1999; Luben et al. 2008). The metabolism of different DBP species varies (IPCC 2000), so it is insufficient to analyze DBPs as a whole or to use TTHM as a proxy. Investigation of the relation between non-THM by-products and reproductive outcomes is required to understand whether any specific DBP may cause health effects. A detailed assessment of the DBP mixture is required to provide an understanding of any observed results.

Sample sizes have often been insufficient to produce stable results, and this may have resulted in chance findings and mixed results, although there are exceptions. For example, the studies by Hwang et al. (2002, 2008) and Nieuwenhuijsen et al. (2008) have provided sufficiently large numbers of cases to create various exposure categories with more stable risk estimates, and were able to examine exposure–response relationships to some extent. A related limitation of the presented meta-analyses is the relatively small number of studies and therefore the need to conduct relatively simple analyses, comparing high-versus low-exposure categories and combining what could be regarded as different and/or inconsistent estimates of exposure (e.g., high vs. low TTHM concentration and chlorination vs. nonchlorination). For instance, in Table 2, for all congenital anomalies Chisholm et al. (2008) used cut points of < 60 μg/L TTHM for low exposure, whereas the high-exposure group used by Hwang et al. (2008) was > 20 μg/L TTHM. Although we assume that the meta-analysis is statistically accurate, the biological basis for comparing studies with this degree of heterogeneity in the definition of exposure is more problematic, and this should be taken into account when interpreting the results. Ideally, all the exposure categories have the same cutoffs, but in practice, this is often impossible because of the different local conditions. We also performed sensitivity analyses leaving out the studies with qualitative estimates (e.g., chlorination vs. nonchlorination), but it generally did not materially change the summary risk estimates, except for nervous system defects including NTDs [from 1.06 (95% CI, 0.89–1.26) to 1.28 (95% CI, 0.89–1.83)] and cleft palate only [from 1.03 (95% CI, 0.89–1.19) to 1.14 (95% CI, 0.80–1.62)], but they remained not statistically significant.

The analyses of TTHM exposure–response relationships combined more comparable exposure levels and were therefore probably more informative, but they could only be conducted for a few end points because of the lack of a sufficient number of studies. The question here is whether TTHM may be the putative agent or a (not so good) marker for something else. Also, as a result of the small number of cases in the studies and to increase power, congenital anomalies have often been categorized into main groups such as NTDs, major heart defects, and abdominal defects, or as all congenital anomalies combined. These anomalies, however, are generally heterogeneous with respect to both phenotype and presumed etiology, and combining them may not be appropriate. For example, Nieuwenhuijsen et al. (2008) showed that focusing on isolated subcategories may result in different findings. Of course, this was possible only because of the large number of subjects in the study.

The retrospective and registry-based nature of many of the studies has meant that information on lifestyle factors, such as maternal smoking and alcohol consumption, has often been lacking. These factors are not necessarily potential confounders, as it seems unlikely that levels of chlorination by-products are related to such factors, nor are they established risk factors for all congenital anomalies. However, behavioral patterns such as ingestion of tap water may be associated with these factors (Forssén et al. 2007, 2008). Furthermore, registry completeness is a factor that may make studies incomparable. In some places, case ascertainment occurs immediately after birth, so some malformations may not be recorded, especially malformations such as hypospadias and cardiac defects, where the less-severe forms may not immediately be recognized. In Norway and Taiwan, birth defects reported up to 7 days after birth were included in the study (Hwang et al. 2008; Magnus et al. 1999), whereas in California (Shaw et al. 2003) and Sweden (Cedergren et al. 2002), cases were diagnosed up to 1 year after birth. However, we anticipate that random underreporting would weaken any observed association rather than introduce a spurious effect. A further discrepancy between studies was whether the study population had been extended beyond live births. In the European Union, terminations accounted for 18% and fetal deaths for 2% of congenital anomalies [European Surveillance of Congenital Anomalies (EUROCAT) 2005]. However, whereas some study populations included terminations and stillbirths (i.e., Dodds et al. 1999; Klotz and Pyrch 1999; Nieuwenhuijsen et al. 2008), others did not (i.e., Cedergren et al. 2002; Chisholm et al. 2008; Hwang et al. 2008).

The strongest, albeit relatively modest, evidence for a possible association with DBPs was found for one specific type of cardiac defect: ventricular septal defect. This finding is of interest in light of studies of other environmental exposures in which associations for this particular birth defect have been observed with, for example, air pollutants (Gilboa et al. 2005; Ritz et al. 2002). However, ventricular septal defects are also among the anomalies that are most subject to variable diagnosis and reporting in routine anomaly registries; all three studies that reported increased risks of ventricular septal defects in relation to high chlorination exposure were based on large, regional or nationwide, congenital anomaly registries in which variable reporting is common (Rankin et al. 2005) and may affect geographic comparison studies such as the DBP studies.

Only one study (Shaw et al. 2003) has examined gene–environment interaction and/or the presence of susceptible groups, and this study did not find any effect. There is some evidence, however, to suggest that cytochrome P450 2E1 (CYP2E1) or glutathione *S*-transferase T1 may play a role in the susceptibility to DBPs, and this should be further explored (Infante-Rivard 2004; Infante-Rivard et al. 2002).

Animal and cell studies have found some effects associated with DBPs. NTDs and craniofacial defects have been found with administration of di/trichloroacetic acid in rats (Smith et al. 1989a), and cardiac malformations have been induced at high doses of dichloroacetic acid (Smith et al. 1992). Hunter et al. (1996) observed changes in neural tube development when mouse embryos were exposed to HAAs. Several chloroacetonitrile compounds have been shown to increase the rate of resorption, to reduce fetal body weight and survival (Smith et al. 1997), and to result in an increase in malformations of the cardiovascular, digestive, soft tissue, and urogenital systems (Smith et al. 1988, 1989b). However, these adverse developmental effects have only been demonstrated at high doses in conjunction with severe fetotoxicity. Furthermore, the mechanisms for possible effects are still unclear, although some have been suggested. Low levels of folate have been associated with several congenital anomalies such as neural tube defects (EUROCAT 2003). Alston (1991) found that chloroform inhibited methionine biosynthesis in cell culture. Dow and Green (2000) showed that trichloroacetic acid interacts with vitamin B12, probably by a free radical mechanism, inhibiting both the methylmalonyl coenzyme A and methionine salvage pathways in rats. As a result of the latter, a secondary folate deficiency develops because of the methyl folate trap, leading to a major impairment in formate metabolism. Folate and folic acid are forms of vitamin B that are involved in the synthesis, repair, and functioning of DNA and are required for the production and maintenance of cells (Kamen 1997). Folate plays an important role for cells undergoing rapid turnover, such as tissues in the developing fetus.

## The Way Forward

Given the many studies conducted and the limited evidence of an association between chlorination by-products and congenital anomalies, we might ask whether there is a need to conduct more studies, and if so, what should they look at? Disinfection of drinking water is an important part of public health, and many people are exposed to chlorination by-products not only through ingestion but also through other activities such as showering and bathing. Ongoing surveillance of any possible adverse health effects is therefore warranted, even though the relative risks may be small. As mentioned above, the mixture of the by-products may differ by geographic area and time, for example, because of changes in water treatment methods, and generally only indicator substances such as TTHM have been used to examine the health risks. It is important that we understand the underlying mixture of the by-products, both in existing and new studies, and where possible, we should examine any possible health risks of specific DBPs or mixtures. Studies are needed to examine the potential effects of certain mixtures, such as brominated species, in more specific locations. For example, Perth, Australia (see Chisholm et al. 2008) or the Barcelona area in Spain (see Villanueva et al. 2006) might be suitable places because of their high levels of brominated compounds.

Furthermore, it would be worthwhile to examine the various exposure pathways and routes other than ingestion in more detail, specifically for volatile by-products such as THMs, as the level of exposure and metabolism may be different and the measures for exposure prevention are likely to be different. This can probably only be done in case–control studies; in such studies it would be necessary to estimate exposure in the most critical (early) periods of pregnancy, which could prove difficult. Prospective exposure assessment through a cohort design would be more appropriate, but practical and financial constraints preclude such a study, as the size of the cohort would need to be extremely large to study rare congenital anomalies.

Regarding the outcomes, the focus of future studies should be on subcategories of congenital anomalies, rather than on the whole group, and should focus on anomalies for which the ascertainment is reasonably complete and consistent if registry-based designs are used. Findings for ventricular septal defects should be followed up, preferably in well-designed case–control studies. The study by Nieuwenhuijsen et al. (2008) showed an excess risk for bromoform and gastroschisis; these findings may be worth examining in more detail and in a different population. One of the problems in that study was the low levels of bromoform in England and Wales, and future studies should be conducted in places where bromoform levels are higher (such as Perth).

Further work is needed on the relation between potential confounders such as smoking and alcohol intake, as well as the relation with by-products in the water and personal behavioral characteristics such as tap water ingestion (instead of bottled water), showering, and bathing to examine to what extent confounding may explain findings for registry-based studies where this information is missing.

There is some suggestion that some chlorination by-products may interfere with folate metabolism; this and other potential mechanisms such as oxidative stress and genotoxicity could be examined with biomarkers in pregnant women to assess to what extent this may be possible. Furthermore, genotyping may identify susceptible populations (e.g., those with CYP2E1).

## Figures and Tables

**Table 1:** Summary of epidemiologic studies on chlorinated disinfection by-products and adverse reproductive outcomes.

Reference	Study details	Cases	Sample population	Exposure assessment	Other risk factors included
Aschengrau et al. 1993	Massachusetts, USA Two hospitals 1977–1980 1,039 major congenital malformations, urinary tract defects, respiratory tract defects; 1,177 controls	1,039 major congenital anomalies, urinary tract defects, respiratory tract defects	Ongoing population-based study of 14,130 obstetrics patients	Based on maternal residential address to ascertain type of water supply, chlorination vs. chloramination, and ground/mixed water vs. surface water	Maternal age, pregnancy history, alcohol, ethnicity, hospital payment, other water contaminants
Bove et al. 1995	New Jersey, USA 75 towns with a public water supply 1985–1988 Sample population: 81,602	All birth defects, 669 surveillance; 118 central nervous system defects; 83 oral cleft defects; 56 NTDs; 108 major cardiac defects	From live birth and fetal death (> 20 weeks) registries	Based on maternal residential address and municipal water surveys to estimate monthly THM levels (five or six exposure categories)	Maternal age, ethnicity, sex of baby, primipara, prenatal care, education, previous stillbirth or miscarriage, other contaminants
Dodds et al. 1999	Nova Scotia, Canada 1988–1995 study population: 49,842 births	77 NTDs, 82 cleft defects, 430 major cardiac defects, 197 stillbirths, 96 chromosomal abnormalities	Live and still birth registry and fetal anomaly database	Based on maternal residential address and TTHM levels for public water facilities (three sampling locations) modeled using linear regression on the basis of observations by year, month, and facility (four exposure categories)	Maternal age, parity, maternal smoking, attendance prenatal classes, neighborhood, family income, sex of baby, pregnancy and predelivery weight Adjusted: NTDs, cardiac defects, chromosomal abnormalities, income
Dodds and King 2001	Nova Scotia, Canada 1988–1995 Study population: 49,842 births	77 NTDs, 430 cardiovascular anomalies, 85 cleft defects, 96 chromosomal abnormalities	Live and still birth registry and fetal anomaly database	Based on maternal residential address and TTHM, chloroform, and BDCM levels for public water facilities (three sampling locations) modeled using linear regression on the basis of observations by year, month, and facility (four exposure categories) (*r*= 0.44 for TTHM and BDCM)	Maternal age, parity, maternal smoking, attendance prenatal classes, neighborhood, family income, sex, pregnancy and predelivery weight Adjusted: NTDs, cardiac defects, cardiovascular; chromosomal; maternal age and income; Cleft: maternal age
Klotz and Pyrch 1999	New Jersey, USA 1993–1994 Sample population: all births, of which 112 cases and 248 controls were selected	112 NTDs	Birth registry and birth defects and fetal death (> 20 weeks) registry	Based on residential address and public water facility TTHM data, and tap-water sampling for TTHM, HANs, and HAAs (three to five exposure categories)	Sociodemographics: pregnancy and premedical history, parental occupation, use of vitamins
Magnus et al. 1999	Norway Study population: 141,077	2,608 all birth defects, 62 NTDs, 250 major cardiac defects, 91 respiratory defects, 122 urinary defects	Birth registry (≥ 16 weeks)	Chlorination yes vs. no Color high vs. low (in chlorinated water average TTHM = 9.4 μg/L , average HAAs = 14.6 μg/L)	Maternal age, parity, geographical placement, population density, industry profile
Källen and Robert 2000	Sweden (1985–1994) Sample population: no chlorination 74,324 singletons Na-hypochlorite: 27,731 singletons chlorine dioxide: 15,429 singletons	Congenital anomalies, including NTDs: 7 anencephaly, 61 spina bifida, 35 hydrocephaly, 1,108 cardiac defects, 223 facial cleft defects, 213 hypospadias	Birth registry, congenital malformation registry, child cardiology registry, and cytogenetic registry	No vs. sodium hypochlorite (no vs. chlorine dioxide)	Year of birth, maternal age, parity, maternal education, maternal smoking, congenital malformation, and childhood cancer, maternal age, year of birth
Cedergren et al. 2002	Sweden 1982–1996 Study population: 59,422	753 cardiac defects	Birth registry	> 10 mg/L vs. ≤ 10 mg/L TTHM in surface water; hypochloride and chlorine dioxide vs. hypochloride in surface water	Maternal age, parity, smoking, education
Hwang et al. 2002	Norway 1993–1998 Sample population: 285,631	5,764 any birth defect, 138 NTDs, 46 anencephalus, 81 spina bifida, 68 hydrocephalus, 537 cardiac defects, 279 ventricular septal defects, 73 atrial septal defects, 192 respiratory defects, 343 oral cleft defects, 95 cleft palate, 67 cleft lip, 232 urinary tract defects, 102 obstructive urinary tract defects	Birth registry (> 16 weeks)	Chlorination (yes/no) and level of water color (mg Pt/L: > 10, 10–19.9, ≥ 20)	Maternal age, parity, socioeconomic status, centrality, population density
Shaw et al. 2003	California, USA Study 1: 538 NTD cases and 539 controls selected California, USA Study 2: 265 NTD cases, 207 conotruncal heart defect cases, 109 orofacial cleft cases, 481 controls	Study 1: 538 NTDs (anencephaly and spina bifida) Study 2: 265 NTDs (anencephaly and spina bifida), 207 conotruncal heart defects, 481 orofacial clefts	Live births, fetal deaths, and terminations	Studies 1 and 2: continuous TTHM categorical: 0, 1–24, 25–49, 50–74, and ≥ 75 μg/L TTHM Also Study 1: ≥ 50 vs. < 50 μg/L and > 5 glasses ≥ 50 vs. < 50 μg/L and > 5 glasses Study 1: chloroform ≥ 13.2 vs. < 12.2 μg/L BDCM ≥ 4.2 vs. < 4.2 μg/L CDBM ≥ 1.7 vs. < 1.7 μg/L Study 2: chloroform ≥ 15.0 vs. < 15.0 μg/L BDCM ≥ 9.6 vs. < 9.6 μg/L CDBM ≥ 3.6 vs. < 3.6 μg/L MTHFR genotype	Ethnicity, education, body mass index, use of vitamins
Nieuwenhuijsen et al. 2008	England and Wales 2,605,226	Congenital anomalies, 1,434 respiratory (ICD-10 codes Q30–Q34), 8,809 major cardiac (Q20–Q28), 5315 urinary (Q60–Q64), 2,267 abdominal wall (Q79), 3,334 NTDs (Q00, Q01, and Q05), 3,736 cleft lip and palate (Q35–Q37)	Birth and stillbirth registries and national and local congenital anomalies registries	THMs	Maternal age, deprivation, sex
Chisholm et al. 2008	Perth, Australia 2000–2004 20,870 live births	1,097 congenital anomalies, 59 nervous system defects (BPA 74,000–74,299), 260 cardiovascular defects (BPA 74,500–74,299), 17 respiratory system defects (BPA 74,800–74,899), 101 gastrointestinal defects (BPA 74,900–75,199), 351 urogenital defects (BPA 75,200–75,399), 282 musculoskeletal defects (BPA 75,400–75,699), 36 congenital anomalies of integument (BPA 75,700–75,799) (*n*= 1,097)	Western	TTH	Maternal age
Luben et al. 2008	Arkansas, USA 1998–2002 320 cases and 614 controls, and a subset of 40 cases and 243 controls	320 and 40 hypospadias	Birth and birth defect registry from Arkansas Reproductive Health Monitoring System and National Birth Defects Prevention Study	THMs and HAAs, and personal characteristics in the subset	Maternal and paternal age, race, and education, marital status, maternal use of alcohol and tobacco, parity, prenatal care, 1- and 5-min Apgar scores, birth weight, method of delivery, any risks, procedures, or complications associated with delivery
Hwang et al. 2008	Taiwan 2001–2003 396,049 births	2,148 congenital anomalies, including 43 anencephalus (ICD-9 code 740.0), 118 hydrocephalus (code 741.0), 59 ventricular septal defects (code 745.4), 19 atrial septal defects (code 745.5), 24 tetralogy of Fallot (code 745.2), 358 cleft palate or lips (code 749.0), cleft lip (code 749.1), 76 renal agenesis and dysgenesis (code 753.0), 49 obstructive urinary tract defects (code 753.2), 72 hypospadias (code 752.61), 166 chromosome anomalies (code 758)	Birth registry	High (TTHM 20 + μg/L), medium (TTHM 10–19 μg/L), low exposure (TTHM 5–9 μg/L), and 0–4 μg/L	Sex of infant, maternal age (< 20 years; 20–34 years; ≥ 35 years), plurality (singleton and multiple birth), maternal health status, population density
Iszatt N, Nieuwenhuijsen MJ, Toledano MB, Nelson P, Elliott P, unpublished data	Southeast England 1997–1998 363 hypospadias cases and 346 population controls	363 hypospadias	Population based	THMs	Income, birth weight, folate supplement use during pregnancy, maternal smoking, maternal occupational exposure to phthalates

Abbreviations: BPA, British Paediatric Association Classification of Disease (BPA 1979); CDBM, chlorodibromomethane; ICD-9, International Classification of Diseases, 9th Revision [World Health Organization (WHO) 1977]; ICD-10, International Classification of Diseases, 10th Revision (WHO 1992).

**Table 2:** Summary of meta-analyses of epidemiological studies on chlorinated disinfection by-products and adverse reproductive outcomes.

Outcome	Exposure	Studies included	*p*-Value of test for heterogeneity	Egger test; weighted *p*-value for intercept	Summary estimate
Any congenital anomaly	High vs. low chlorination by-products	a,b,i,l,n	0.041	0.16	1.17 (1.02–1.34)
Nervous system defects including neural tube defects	High vs. low chlorination by-products	b,c,e,i,j,k,l,m	0.058	0.11	1.06 (0.89–1.26)
Nervous system defects including neural tube defects	Per 10 μg/L TTHM	b,c,e,j,k,l,m	0.46	0.20	1.01 (0.95–1.06)
Nervous system defects including neural tube defects	BDCM	d,j,k	0.005	0.12	1.15 (0.59–2.25)
Anencephalus	High vs. low chlorination by-products	i,j,k,o	0.45	0.17	1.48 (0.92–2.39)
Hydrocephalus	High vs. low chlorination by-products	g,i,o	0.18	0.54	0.92 (0.57–1.48)
Spina bifida	High vs. low chlorination by-products	g,i,j,k,l	0.35	0.55	1.22 (0.76–1.97)
Major cardiac defects	High vs. low chlorination by-products	b,c,g,h,i,k,l,m	0.017	0.27	1.16 (0.98–1.37)
Major cardiac defects	Per 10 μg/L TTHM	b,c,k,l,m	0.49	0.39	0.99 (0.95–1.04)
Ventricular septal defects	High vs. low chlorination by-products	i,l,o	0.69	0.13	1.59 (1.21–2.07)
Respiratory defects	High vs. low chlorination by-products	a,i,l,m	0.064	0.29	1.12 (0.91–1.37)
Oral cleft or cleft palate defects	High vs. low chlorination by-products	a,b,c,g,i,k,l,o	0.32	0.067	0.98 (0.88–1.08)
Oral cleft or cleft palate defects	Per 10 μg/L TTHM	b,c,k,l,o	0.44	0.26	1.00 (0.96–1.05)
Cleft palate only	High vs. low chlorination by-products	i,k,l,o	0.26	0.53	1.03 (0.89–1.19)
Urinary tract defects	High vs. low chlorination by-products	a,i,l,m	0.012	0.002	1.33 (0.92–1.92)
Obstructive urinary defects	High vs. low chlorination by-products	i,l,o	0.37	0.12	1.07 (0.87–1.30)
Hypospadias	High vs. low chlorination by-products	g,n,o,p	0.20	0.17	1.03 (0.84–1.28)

Summary estimates are shown as OR (95%CI).

a Aschengrau et al. (1993).

b Bove et al. (1995).

c Dodds et al. (1999).

d Dodds and King (2001).

e Klotz and Pyrch (1999).

f Magnus et al. (1999).

g Källen and Robert (2000).

h Cedergren et al. (2002).

i Hwang et al. (2002).

j Study 1, Shaw et al. (2003).

k Study 2, Shaw et al. (2003).

l Nieuwenhuijsen et al. (2008).

m Chisholm et al. (2008).

n Luben et al. (2008).

o Hwang et al. (2008).

p Iszatt N, Nieuwenhuijsen MJ, Toledano MB, Nelson P, Elliott P (unpublished data).

## References

[b1-ehp-117-1486] Alston TA (1991). Inhibition of vitamin B12-dependent methionine biosynthesis by chloroform and carbon tetrachloride. Biochem Pharmacol.

[b2-ehp-117-1486] Aschengrau A, Zierler S, Cohen A (1993). Quantity of community drinking water and the occurrence of late adverse pregnancy outcomes. Arch Environ Health.

[b3-ehp-117-1486] Bove FJ, Fulcomer MC, Klotz JB, Esmart J, Dufficy EM, Savrin JE (1995). Public drinking water contamination and birth outcomes. Am J Epidemiol.

[b4-ehp-117-1486] Bove F, Shim Y, Zeitz P (2002). Drinking water contaminants and adverse pregnancy outcomes: a review. Environ Health Perspect.

[b5-ehp-117-1486] BPA (1979). British Paediatric Association Classification of Disease.

[b6-ehp-117-1486] Cedergren MI, Selbing AJ, Lofman O, Källen B (2002). Chlorination byproducts and nitrate in drinking water and risk of congenital cardiac defects. Environ Res.

[b7-ehp-117-1486] Chisholm K, Cook A, Bower C, Weinstein P (2008). Risk of birth defects in Australian communities with high brominated disinfection by-product levels. Environ Health Perspect.

[b8-ehp-117-1486] Cochran WG (1954). The combination of estimates of different experiments. Biometrics.

[b9-ehp-117-1486] DerSimonian R, Laird N (1986). Meta-analysis in clinical trails. Control Clin Trials.

[b10-ehp-117-1486] Dodds L, King W, Woolcott C, Pole J (1999). Trihalomethanes in public water supplies and adverse birth outcomes. Epidemiology.

[b11-ehp-117-1486] Dodds L, King WD (2001). Relation between trihalomethane compounds and birth defects. Occup Environ Med.

[b12-ehp-117-1486] Dow JL, Green T (2000). Trichloroethylene induced vitamin B12 and folate deficiency leads to increased formic acid excretion in the rat. Toxicology.

[b13-ehp-117-1486] Egger M, Davey Smith G, Schneider M, Minder C (1997). Bias in meta-analysis detected by a simple, graphical test. BMJ.

[b14-ehp-117-1486] EUROCAT (European Surveillance of Congenital Anomalies) (2003). EUROCAT Special Report: Prevention of Neural Tube Defects by Periconceptional Folic Acid Supplementation in Europe.

[b15-ehp-117-1486] EUROCAT (European Surveillance of Congenital Anomalies) (2005). WHO Collaborating Centre for the Epidemiologic Surveillance of Congenital Anomalies Report 2004–2005.

[b16-ehp-117-1486] Forssén UM, Herring AH, Savitz DA, Nieuwenhuijsen MJ, Murphy PA, Singer PC (2007). Predictors of use and consumption of public drinking water among pregnant women. J Expo Sci Environ Epidemiol.

[b17-ehp-117-1486] Forssén UM Wright JM Herring AH Savitz DA Nieuwenhuijsen MJ Murphy PA 2008Validity and predictors of changes in water use during pregnancyJ Expo Sci Environ Epidemiol10.1038/jes.2008.59 [Online 1 October 2008] 18830235

[b18-ehp-117-1486] Gevecker Graves C, Matanoski GM, Tarfdiff RG (2001). Weight of evidence for an association between adverse reproductive and developmental effects and exposure to disinfection by-products: a critical review. Regul Toxicol Pharmacol.

[b19-ehp-117-1486] Gilboa SM, Mendola P, Olshan AF, Langlois PH, Savitz DA, Loomis D (2005). Relation between ambient air quality and selected birth defects, seven county study, Texas, 1997–2000. Am J Epidemiol.

[b20-ehp-117-1486] Hunter ES, Roger EH, Schmid JE, Richard A (1996). Comparative effects of haloacetic acids in whole embryo culture. Teratology.

[b21-ehp-117-1486] Hwang B-F, Jaakkola JJK (2003). Water chlorination and birth defects: a systematic review and meta-analysis. Arch Environ Health.

[b22-ehp-117-1486] Hwang B-F, Jaakkola JJK, Guo HR (2008). Water disinfection byproducts and the risk of specific birth defects: a population-based cross-sectional study in Taiwan. Environ Health.

[b23-ehp-117-1486] Hwang B-F, Magnus P, Jaakkola JJK (2002). Risk of specific birth defects in relation to chlorination and the amount of natural organic matter in the water supply. Am J Epidemiol.

[b24-ehp-117-1486] Infante-Rivard C (2004). Drinking water contaminants, gene polymorphisms, and fetal growth. Environ Health Perspect.

[b25-ehp-117-1486] Infante-Rivard C, Amre D, Sinnett D (2002). GSTT1 and CYP2E1 polymorphisms and trihalomethanes in drinking water: effect on childhood leukemia. Environ Health Perspect.

[b26-ehp-117-1486] IPCS (International Programme on Chemical Safety) (2000). Disinfectants and Disinfectant By-products Environmental Health Criteria 216.

[b27-ehp-117-1486] Källen BAJ, Robert E (2000). Drinking water chlorination and delivery outcome—a registry-based study in Sweden. Reprod Toxicol.

[b28-ehp-117-1486] Kamen B (1997). Folate and antifolate pharmacology. Semin Oncol.

[b29-ehp-117-1486] Key J, Hodgson S, Omar RZ, Jensen TK, Thompson SG, Boobis AR (2006). Meta-analysis of studies of alcohol and breast cancer with consideration of the methodological issues. Cancer Causes Control.

[b30-ehp-117-1486] Klotz JB, Pyrch LA (1999). Neural tube defects and drinking water disinfection by-products. Epidemiology.

[b31-ehp-117-1486] Kuivinen J, Johnsson H (1999). Determination of trihalomethanes and some chlorinated solvents in drinking water by head-space technique with capillary column gas-chromatography. Water Res.

[b32-ehp-117-1486] Light RJ, Pillemer DB (1984). Summing up: The Science of Reviewing Research.

[b33-ehp-117-1486] Luben TJ, Nuckols JR, Mosley BS, Hobbs C, Reif JS (2008). Maternal exposure to water disinfection by-products during gestation and risk of hypospadias. Occup Environ Med.

[b34-ehp-117-1486] Magnus P, Jaakkola JJK, Skrondal A, Alexander J, Becher G, Krogh T (1999). Water chlorination and birth defects. Epidemiology.

[b35-ehp-117-1486] Mantel N, Haenszel W (1959). Statistical aspects of the analysis of data from retrospective studies of disease. J Natl Cancer Inst.

[b36-ehp-117-1486] National Center for Biotechnology Information (2009). PubMed.

[b37-ehp-117-1486] Nieuwenhuijsen MJ, Toledano MB, Bennett J, Best N, Hambly P, de Hoogh K (2008). Chlorination disinfection by-products and risk of congenital anomalies in England and Wales. Environ Health Perspect.

[b38-ehp-117-1486] Nieuwenhuijsen MJ, Toledano MB, Eaton NE, Elliott P, Fawell J (2000b). Chlorination disinfection by-products in water and their association with adverse reproductive outcomes: a review. Occup Environ Med.

[b39-ehp-117-1486] Nieuwenhuijsen MJ, Toledano MB, Elliott P (2000a). Uptake of chlorination disinfection by-products; a review and a discussion of its implications for epidemiological studies. J Expos Anal Environ Epidemiol.

[b40-ehp-117-1486] R Project for Statistical Computing (2009). What Is R?.

[b41-ehp-117-1486] Rankin J, Pattenden S, Abramsky L, Boyd P, Jordan H, Stone D (2005). Prevalence of congenital anomalies in five British regions, 1991–99. Arch Dis Child Fetal Neonatal Ed.

[b42-ehp-117-1486] Reif JS, Hatch MC, Bracken M, Holmes LB, Schwetz BA, Singer PC (1996). Reproductive and development effects of disinfection by-products in drinking water. Environ Health Perspect.

[b43-ehp-117-1486] Richardson S, Meyers RA (1998). Drinking water disinfection by-products. In: Encyclopedia of Environmental Analysis and Remediation. Encyclopedia of Environmental Analysis and Remediation.

[b44-ehp-117-1486] Richardson SD, Plewa MJ, Wagner ED, Schoeny R, DeMarini DM (2007). Occurrence, genotoxicity, and carcinogenicity of emerging disinfection by-products in drinking water: a review and roadmap for research. Mutat Res.

[b45-ehp-117-1486] Ritz B, Yu F, Fruin S, Chapa G, Shaw GM, Harris JA (2002). Ambient air pollution and risk of birth defects in Southern California. Am J Epidemiol.

[b46-ehp-117-1486] Rook JJ (1974). Formation of haloforms during chlorination of natural waters. J Soc Water Treat Exam.

[b47-ehp-117-1486] Shaw GM, Ranatunga D, Quach T, Neri E, Correa A, Neutra R (2003). Trihalomethane exposures from municipal water supplies and selected congenital malformations. Epidemiology.

[b48-ehp-117-1486] Smith MK, George EL, Zenick H, Manson JM, Stober JA (1987). Developmental toxicity of halogenated acetonitriles: drinking water by-products of chlorine disinfection. Toxicology.

[b49-ehp-117-1486] Smith MK, Randall JL, Read JL, Stober JA (1992). Developmental toxicity of dichloroacetate in the rat. Teratology.

[b50-ehp-117-1486] Smith MK, Randall JL, Stober JA (1989a). Teratogenic activity of trichloroacetic acid in the rat. Teratology.

[b51-ehp-117-1486] Smith MK, Randall JL, Stober JA, Read EJ (1989b). Developmental toxicity of dichloroacetonitrile: a by-product of drinking water disinfection. Fundam Appl Toxicol.

[b52-ehp-117-1486] Smith MK, Randall JL, Tocco DR, York RG, Stober JA, Read EJ (1988). Teratogenic effects of trichloroacetonitrile in the Long-Evans rat. Teratology.

[b53-ehp-117-1486] Tardiff RG, Carson ML, Ginevan ME (2006). Updated weight of evidence for an association between adverse reproductive and developmental effects and exposure to disinfection byproducts. Regul Toxicol Pharmacol.

[b54-ehp-117-1486] Villanueva CM, Cantor KP, Grimalt JO, Malats N, Silverman D, Tardon A (2007). Bladder cancer and exposure to water disinfection by-products through ingestion, bathing, showering and swimming in pools. Am J Epidemiol.

[b55-ehp-117-1486] Whitaker H, Nieuwenhuijsen MJ, Best N (2003). The relationship between water chloroform levels and uptake of chloroform: a simulation study. Environ Health Perspect.

[b56-ehp-117-1486] WHO (1977). Manual of the International Statistical Classification of Disease, Injuries, and Causes of Death.

[b57-ehp-117-1486] WHO (1992). International Statistical Classification of Diseases and Related Health Problems.

